# Consumption of Chokeberry Bio-Products Improves Specific Metabolic Parameters and Increases the Plasma Antioxidant Status

**DOI:** 10.3390/antiox13060699

**Published:** 2024-06-07

**Authors:** Ewa Olechno, Anna Puścion-Jakubik, Katarzyna Socha, Caterina Pipino, Małgorzata Elżbieta Zujko

**Affiliations:** 1 Department of Food Biotechnology, Faculty of Health Science, Medical University of Białystok, Szpitalna 37 Street, 15-295 Białystok, Poland; ewa.olechno@sd.umb.edu.pl; 2 Department of Bromatology, Faculty of Pharmacy with the Division of Laboratory Medicine, Medical University of Białystok, Mickiewicza 2D Street, 15-222 Białystok, Poland; katarzyna.socha@umb.edu.pl; 3 Center for Advanced Studies and Technology, G. d’Annunzio University, 66100 Chieti, Italy; caterina.pipino@unich.it

**Keywords:** chokeberry juice, chokeberry fiber, metabolic disorders, hyperglycemia, hypertension, dyslipidemia, inflammation

## Abstract

Because of its high antioxidant activity, chokeberry can be used both in the prevention and treatment of various metabolic disorders. In this study, for the first time, the synergistic effects of chokeberry juice and chokeberry fiber on selected metabolic and anthropometric parameters were assessed during a 90-day intervention including 102 people (67 women and 35 men). After 60 days of intervention with chokeberry juice, statistically significant increases in the muscle mass and antioxidant potential of the serum were observed. In turn, there were decreases in the waist circumference, systolic blood pressure, diastolic blood pressure, heart rate, glycated hemoglobin, glucose, LDL cholesterol, eGFR, and ALT level. The addition of chokeberry fiber for the next 30 days resulted in stabilizations of the diastolic blood pressure, glycated hemoglobin, glucose, and waist circumference, as well as reductions in the values of the heart rate, LDL cholesterol, insulin, and AST level. After 90 days, a significant increase in the FRAP value was also observed. This intervention indicates that chokeberry products may have a beneficial effect on metabolic health and serve as a foundation for developing functional foods.

## 1. Introduction

Nowadays, there is an increase in the incidence of lifestyle-related diseases, including obesity, cardiovascular diseases, liver diseases, neurodegenerative diseases, kidney diseases, and cancer [1]. Oxidative stress, which is associated with the formation of reactive oxygen species, plays an important role in the pathogenesis of these diseases [2]. Reactive oxygen species are produced naturally in the body, including through mitochondrial changes, and can also result from external factors, such as a highly processed diet, chronic stress, injuries, intense physical exercise, alcohol consumption, or cigarette smoking [3]. The disturbance of the antioxidant–oxidant balance leads to damage to bodily cells and, consequently, increases in the risks of many diseases and metabolic disorders [2,3]. The human body has natural mechanisms that protect against the negative effects of oxidative stress. These are enzymatic antioxidants (superoxide dismutase, glutathione peroxidase, and catalase) and non-enzymatic antioxidants (metal-binding proteins, glutathione, uric acid, melatonin, bilirubin, and polyamines) [4]. However, excess reactive oxygen species caused by exogenous factors significantly increase the need for antioxidants supplied in the diet. Dietary factors may significantly influence the increase in the antioxidant potential of the blood plasma. In turn, a high antioxidant potential translates to a better ability of the body to counteract and combat the effects of oxidative stress [5,6]. Natural antioxidants present in food include polyphenol compounds, antioxidant vitamins (vitamins C, E, and A), and trace elements (selenium, zinc, manganese, copper, and iron). These substances participate directly or indirectly in the fight against reactive oxygen species [7,8].

The results of many studies have shown that lifestyle modifications, including a healthy diet rich in antioxidants, are the main therapeutic strategy in the prevention and treatment of chronic diseases [9,10]. In the Polish adult population, a higher intake of dietary antioxidants was significantly associated with higher socioeconomic and health statuses [11]. It was shown that reduced total dietary antioxidants may be considered as an additional risk factor for the development of diabetes and cardiovascular diseases [12,13]. Berries, including chokeberry, are excellent sources of antioxidants. Chokeberry is distinguished by its high antioxidant potential compared to other plant products [14]. It contains many bioactive compounds, including polyphenols, vitamins (among others, vitamin C, vitamin K, and vitamin E), carotenoids, microelements (potassium, magnesium, calcium, and phosphorus), and trace elements (zinc, selenium, manganese, copper, and iron) [15]. Among the polyphenols of chokeberry fruit are flavonoids, especially anthocyanins, as well as procyanidins, flavonols, flavanols, and phenolic acids. Anthocyanins are the main antioxidants of chokeberry fruit. They include cyanidin-3-glucoside, cyanidin-3-galactoside, cyanidin-3-arabinoside, and cyanidin-3-xyloside [16]. Because of their high contents of antioxidant compounds, products made from chokeberry fruit, when regularly consumed, may increase the antioxidant potential of the blood plasma, which is confirmed by literature reports [17]. Additionally, it has been shown that the consumption of chokeberry juice may have hypotensive, lipid-lowering, and hypoglycemic effects, as well as reduce the levels of liver enzymes [18,19,20]. Studies on animals or cell cultures report chokeberry’s hepatoprotective [21,22], anti-inflammatory [23,24,25], neuroprotective [26,27], antidepressant [28], anti-aging [29], anticancer [30], antiviral [31], and antibacterial effects [32]. Because of their contents of valuable health-promoting ingredients, chokeberry products can be used both in the prevention and support of the treatment for existing metabolic disorders [18]. However, many of these issues require confirmation, and more studies involving humans are needed. Previous research has been based on assessing the impacts of the consumption of both chokeberry juice and chokeberry extracts on various health parameters; however, the impacts on some parameters are still unclear and require further investigation.

The aim of this study was to assess the impacts of a dietary intervention using organic chokeberry products (100% chokeberry juice and chokeberry fiber) on specific blood parameters and the antioxidant potential of the plasma. For the first time, the synergistic effect of these two raw materials was examined, as well as the comprehensive assessment of the synergistic effects on selected metabolic and anthropometric parameters.

## 2. Materials and Methods

### 2.1. Recruitment of Participants

We conducted this dietary intervention study among the Polish population. Volunteer participants expressed their willingness to participate in the study by completing the online application form (using Google forms). The inclusion criteria for the study were ages 30–65 and low or moderate physical activity levels.

Exclusion criteria were diabetes (all types); gastric ulcer; acute or chronic gastritis; intestinal diseases, including inflammatory bowel diseases and functional intestinal disorders; taking hypoglycemic, lipid-lowering, immunosuppressive, anticoagulant, or antihypertensive drugs; the use of steroid therapy; high physical activity levels; pregnancy; and breastfeeding.

**Figure 1:**
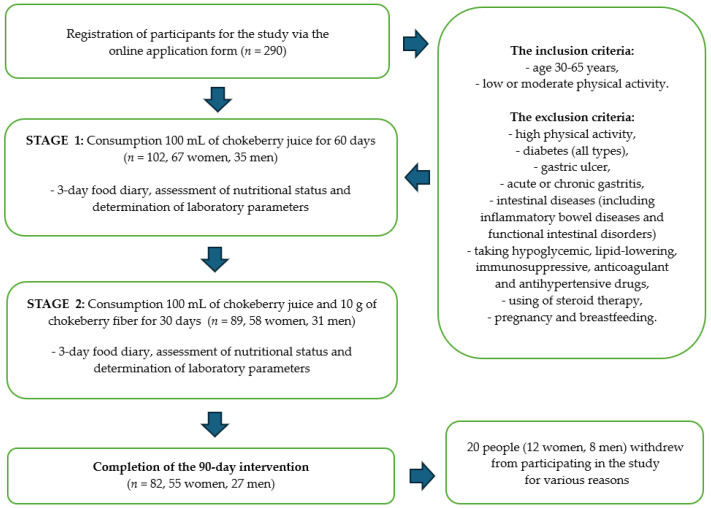
Scheme of the intervention with chokeberry juice and fiber.

Finally, 290 people expressed their willingness to participate in the study. After we considered the data indicated in the surveys and analyzed the exclusion criteria, 102 people (67 women and 35 men) were classified to participate in the study. Twenty people withdrew from participating in the study for various reasons. One of the declared reasons for resignation was the sour taste of the chokeberry products, which was not accepted by all the study participants. Scheme of the intervention is shown on Figure 1.

The dietary intervention lasted for a total of 90 days. Study participants consumed 100 mL of chokeberry juice daily for 60 days. After 2 months, the participants additionally supplemented with chokeberry fiber (10 g/day). Chokeberry juice and fiber were selected based on previous analyses confirming the quality and safety of the raw materials, which we described in detail in our previous publication [33,34]. Chokeberry juice and chokeberry fiber came from ecological cultivation (Poland). This study has the status of a clinical trial—number: NCT06435130.

Qualitative and quantitative assessments of the diet, the assessment of the nutritional status, and the determination of laboratory parameters were performed three times during the study: before the start of the dietary intervention, after 2 months of consuming chokeberry juice (60 days), and after 3 months (next 30 days), i.e., after the monthly consumption of chokeberry juice and fiber together. The characteristics of the study participants are presented in Table 1.

### 2.2. Assessment of Diet and Nutritional Status

A quantitative assessment of the diet was carried out among the study participants according to a 3-day food diary. The diary was based on 2 working days and 1 day off. The participants underwent anthropometric measurements: body height (expressed in centimeters), bodyweight (in kilograms), and waist and hip circumferences (in centimeters).

The participants were asked to keep the diet the same in each of the three stages to avoid interference from other dietary components. Dietary data (caloric intake, protein intake, fat intake, and carbohydrate intake) are presented in Table 2. Height and bodyweight were measured using a growth meter and a body composition analyzer. Body composition analysis assessed the mass and percentage of the fat tissue, the volume of the visceral fat tissue, and the mass of the muscle tissue. BMI was assessed based on these measurements. Additionally, body composition analysis was performed using the bioelectrical impedance method and an InBody 720 device (Biospace, Eonju-ro, Republic of Korea).

### 2.3. Assessment of Metabolic Disorder Parameters

Participants who qualified for the study had specific biochemical parameters tested: fasting glucose concentration, fasting insulin concentration, lipid profile (triglycerides, total cholesterol, LDL cholesterol, and HDL cholesterol), glycated hemoglobin, uric acid, creatinine, estimated glomerular filtration rate (e-GFR), C-reactive protein (CRP), creatinine, and liver enzymes (alanine transaminase (ALT), aspartate transaminase (AST), gamma-glutamylotranspeptydase (GGTP), and homocysteine). The assessment was performed by a certified external laboratory.

The study participants also had their blood pressure and heart rate measured using a blood pressure monitor (twice, at a 5 min interval, and on the non-dominant arm).

### 2.4. Assessment of Ferric-Ion-Reducing Antioxidant Potential (FRAP) in Blood Serum

Additionally, the antioxidant potential of the participants’ blood serum was assessed at each stage of the study using the ferric-ion-reducing antioxidant potential method (FRAP).

The ferric-ion-reducing antioxidant potential (FRAP) was determined according to Benzie and Strain’s method [35] using a Shimadzu UV spectrophotometer (Shimadzu, Kyoto, Japan). This method is based on the reduction of Fe^3+^ ions in the form of a complex with 2,4,6-tri(2-pyridyl)-s-triazine (TPTZ)—TPTZ-Fe^3+^ to Fe^2+^ ions—TPTZ-Fe^2+^ in the presence of antioxidants in samples. The absorbance (an intense blue color) was measured, at 593 nm after 4 min of incubation at 37 °C, against a blank without a sample. The FRAP was calculated from the standard curve and expressed as millimoles per liter.

### 2.5. Assessment of the Compositions of Chokeberry Juice and Fiber

The compositions of the juice and fiber used for the intervention were assessed. The following parameters were determined: antioxidant potential (FRAP), total content of polyphenols (TPC), total flavonoids, total anthocyanins, vitamin C, and elements (Cu, Fe, Mg, Mn, Se, and Zn). Each parameter was determined in three repetitions.

Measurements of antioxidant properties were performed using a UV spectrophotometer (Shimadzu, Kyoto, Japan). Before that, chokeberry juice was thinned with ultrapure water, and chokeberry fiber was extracted with methanol and acetone according to the method in a study by Zujko et al. (2011) [36]. In turn, the contents of the elements were assessed by the microwave mineralization of the samples and then analyzed using the atomic absorption spectrometry (AAS) method [37]. Composition of chokeberry juice and fiber used for the dietary intervention is shown on Table 3.

#### 2.5.1. Assessment of the Total Anthocyanin Content

The Giusti and Wrolstad method [38] was used to assess the anthocyanin content. In this method, samples were incubated (for 15 min at room temperature); then, the absorbance was measured using buffers: chloride (pH = 1.0) and acetate buffer (pH = 4.5). The anthocyanin content was expressed as the cyanidyn-3-glucoside content (mg Cy-3-GL/kg).

#### 2.5.2. Assessment of the Total Flavonoid Content

To assess the content of flavonoids, a method with a mixture of 2% AlCl_2_ and methanol was used. This method involves the creation of aluminum–flavonoid complexes [39]. Then, the samples were incubated (for 10 min at room temperature). After this time, absorbance measurements were made (wavelength: 415 nm). The total flavonoid content was presented as quercetin equivalents (mg QE/kg).

#### 2.5.3. Assessment of the Total Polyphenol Content

The total polyphenol content was determined using the Folin–Ciocalteu reagent [40]. The yellow reagent is oxidized as a result of the reaction with polyphenol components and then reduced in the presence of sodium carbonate—a blue color is created. The resulting samples were incubated (for 30 min at room temperature), and the absorbance was measured (wavelength: 765 nm). The total polyphenol content was expressed as gallic acid equivalents (mg GAE/kg) after calculation from the standard curve.

#### 2.5.4. Assessment of the Ferric-Ion-Reducing Antioxidant Potential (FRAP) in Chokeberry Juice and Chokeberry Fiber

The ferric-ion-reducing antioxidant potentials (FRAPs) of the chokeberry juice and fiber were determined according to Benzie and Strain’s method [35]. The method was the same as in the case of the FRAP assessment of the blood serum. The FRAP contents were expressed as millimoles per kilogram after calculation from the standard curve.

#### 2.5.5. Assessment of the Mineral Contents

Mineralization was carried out to assess the element contents. For this purpose, the samples were weighed (accurate to 1 mg); then, spectrally pure concentrated nitric acid 69% (4.0 mL, 69% HNO_3_, Tracepur, Merck, Darmstadt, Germany) was added. The mineralization process was carried out in a closed-loop microwave system (Speedwave, Berghof, Eningen, Germany). Ultrapure water was used to transfer the obtained samples to the vessels. Ultrapure water was prepared using a Simplicity 185 device (Millipore, Burlington, VT, USA).

The contents of the selected elements in the samples were determined using the AAS method (Z-2000 apparatus, Hitachi, Tokyo, Japan). The mineralizates were diluted with ultrapure water, based on the ranges of the standard curves. The contents of Cu, Mn, and Se were determined by the flameless AAS technique with electrothermal atomization in a graphite cuvette. The determination of the Se content required a palladium–magnesium matrix modifier: Pd concentration—1500 mg/L and Mg concentration—900 mg/L (Merck, Darmstadt, Germany). For the measurement of the Mn, magnesium nitrate (Mg(NO_3_)_2_; concentration: 100 mg/L, Sigma-Aldrich, Merck, Darmstadt, Germany) as a modifier was used. The contents of Fe, Mg, and Zn were determined using the AAS flame technique in an acetylene–air flame with Zeeman background correction. In the case of the Mg content determination, a masking agent was used—1% lanthanum chloride (LaCl_3_, Sigma-Aldrich, Merck, Darmstadt, Germany). To validate this method, a certified reference material was used: tea leaves—INCT–TL–1—a Polish certified reference material for multielement trace analysis (Institute of Nuclear Chemistry and Technology, Warsaw, Poland) [41].

#### 2.5.6. Assessment of the Vitamin C Content

The sum of the L-ascorbic acid and dehydroascorbic acid contents was determined using the high-performance liquid chromatography (HPLC) method with UV detection (wavelength: 254 nm; Perkin Elmer, Waltham, MA, USA). Dehydroascorbic acid was reduced to L-ascorbic acid using dithiothreitol (DTT). The results were expressed in milligrams per kilogram of the product [42].

### 2.6. Consumption of Chokeberry Juice and Fiber

Because of the sour taste of the chokeberry juice, participants could dilute the juice with spring, filtered, or boiled water. The chokeberry fiber could be divided into 2 portions a day (5 g of fiber per serving) and consumed with water or included in a meal. Participants were asked to discontinue the use of antioxidant supplements for at least 7 days prior to laboratory testing and for the duration of the dietary intervention. Additionally, they were advised not to consume larger amounts of chokeberry juice per day than recommended, other juices containing chokeberry, supplements, or other products containing chokeberry fruit.

### 2.7. Statistical Analysis

The obtained results were subjected to statistical analysis using Microsoft Office Excel 2019 and Statistica 13.3 (StatSoft, Tibco, Palo Alto, CA, USA).

The following descriptive statistic parameters were calculated: mean with standard deviation (SD), minimum (Min.) and maximum (Max.), median, and lower (Q1) and upper (Q3) quartiles. The normality of the data distribution was assessed using the following tests: Kolmogorov–Smirnov, Lilliefors, and Shapiro–Wilk tests.

The Wilcoxon paired-order test was used to assess differences between individual groups before and after the intervention. The assessment of the connections between the studied parameters was carried out using Spearman’s correlation. Spearman’s rank correlation coefficients were also determined. The statistically significant level was *p* < 0.05.

## 3. Results

**Figure 2:**
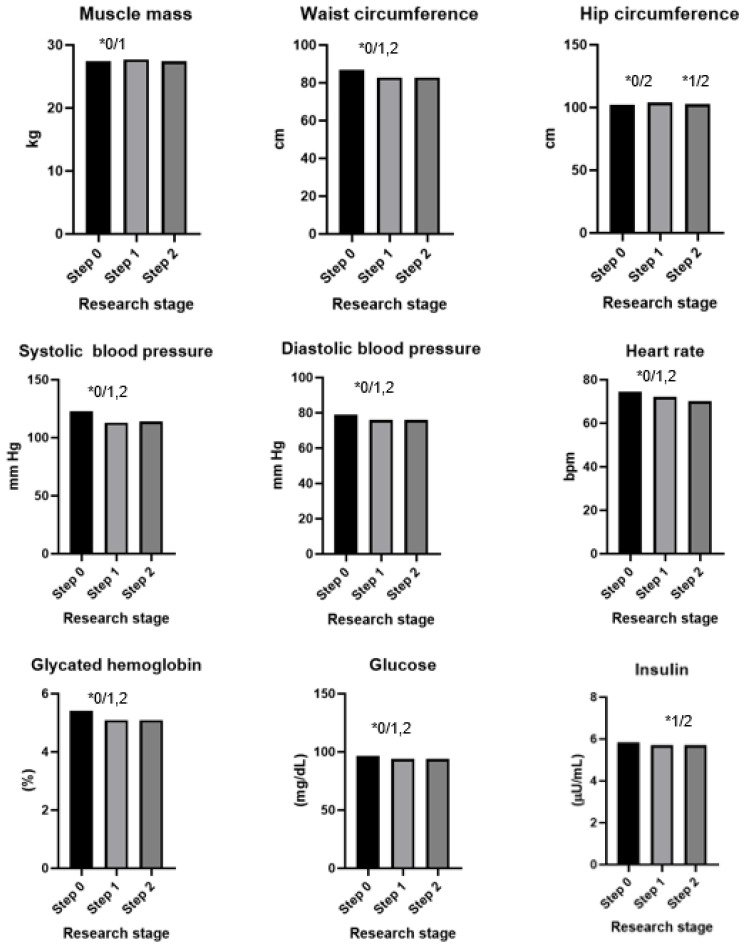

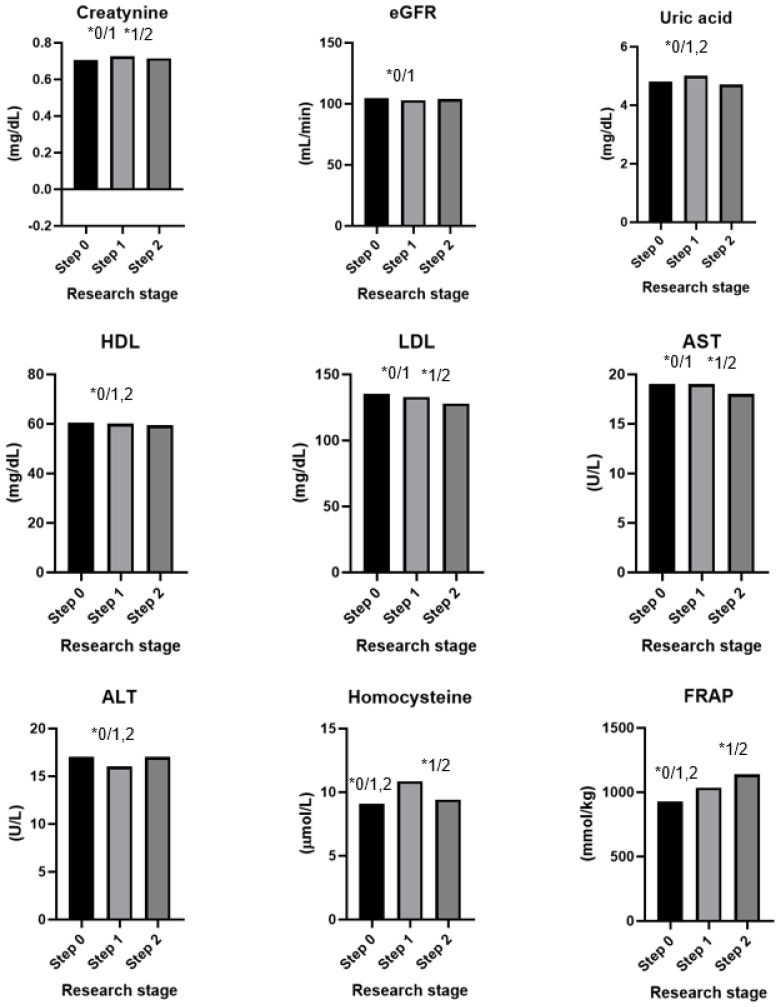
Results of an intervention study involving chokeberry juice and fiber (* *p* < 0.05, 0—step 0, 1—step 1, 2—step 2).

**Figure 3:**
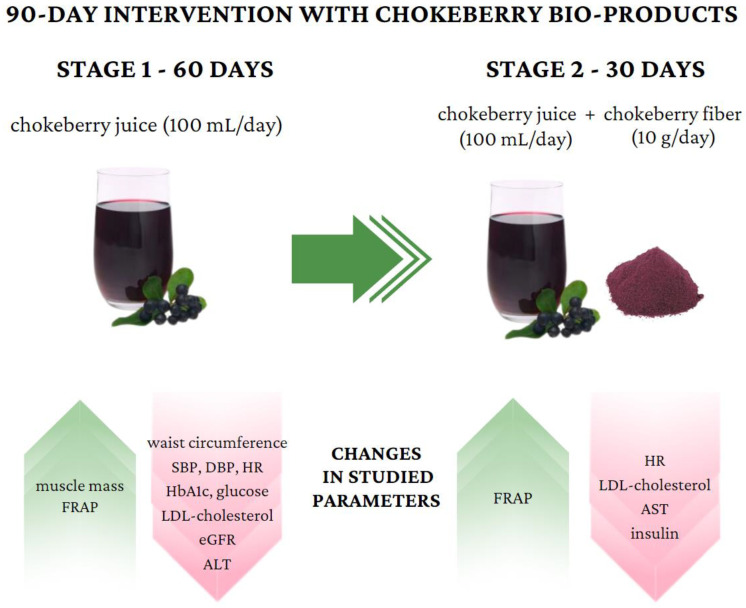
Important changes in studied parameters during chokeberry intervention.

The results of the dietary intervention are presented in Table 4, Figure 2 and Figure 3. After 60 days of intervention with chokeberry juice, statistically significant increases in the muscle mass (median (Q1–Q3): 27.7 kg (24.1–36.2) vs. 27.5 kg (24.1–33.2)) and FRAP (926.02 (808.30–1010.37 mmol/L) vs. 1036.44 (914.22–1168.44 mmol/L)) were observed. The median FRAP parameter of the participants’ diets at stage 0 was 16.78 (11.81–22.99 mmol/kg). The value of this parameter, estimated on the basis of the dietary supply, did not change during the intervention period. An increase in FRAP in the serum resulted from the dietary intervention with chokeberry juice and fiber.

In addition, increases in the levels of creatinine (0.705, 0.630–0.790 mg/dL vs. 0.756, 0.660–0.790 mg/dL), uric acid (4.8, 3.8–5.4 mg/dL vs. 5.0, 4.2–5.6 mg/dL), and homocysteine (9.08, 7.76–10.95 µmol/L vs. 10.87, 9.46–12.60 µmol/L) were observed. A monthly dietary intervention using chokeberry fiber resulted in reductions in the values of these parameters to the following levels: 0.715, 0.660–0.780 mg/dL; 4.7, 4.0–5.5 mg/dL; 9.43, 8.12–11.16 µmol/L.

The intervention of drinking juice for 60 days resulted in decreases in the following parameters: waist circumference (86.8 cm, 80.0–96.0 vs. 82.8, 78.0–92.0 cm), systolic blood pressure (122, 112–130 vs. 113, 105–125 mm Hg), diastolic blood pressure (79, 73–87 vs. 79, 69–83 mm Hg), heart rate (75, 69–82 vs. 72, 66–77 bmp), glycated hemoglobin (5.4, 5.2–5.6 vs. 5.1, 4.9–5.4%), glucose (97, 92–101 vs. 94, 90–97 mg/dL), insulin (5.9, 4.4–7.7 vs. 5.7, 3.9–7.6 µU/mL), LDL cholesterol (136, 108–162 vs. 133, 106–158 mg/dL), ALT (17, 14–25 vs. 16, 12–22 U/L), and e-GFR (105, 98–118 vs. 103, 93–114 mL/min).

The intervention consisting of adding fiber had a bidirectional effect: In the case of some parameters, it stabilized them (waist circumference, hip circumference, diastolic blood pressure, glycated hemoglobin, glucose, and insulin); in the case of others, it additionally reduced or increased them toward favorable values (heart rate, LDL, and AST).

The strongest effect observed was an increase in the antioxidant properties of the plasma, as measured by the FRAP method. The strength of the correlation between the FRAP and the parameter values for which statistically significant differences were found was then assessed. In most cases, the strength of the correlation for the parameters assessed after the 3rd stage was higher than after the second stage. The strongest correlations were found for uric acid, 0.87 and 0.78, respectively, which may indicate a decrease in uric acid levels with prolonged use of chokeberry juice and fiber (Table S1).

## 4. Discussion

In the studies conducted so far, chokeberry was tested both in the form of juice and in the form of a supplement, which could be crucial in understanding its effects. Natural chokeberry products have variable compositions, despite the same forms. Differences in the contents of various nutrients will depend on the variety of the fruit used for production, the cultivation method, soil and weather conditions, the harvest time, as well as the storage and processing of the fruit at later production stages [43,44,45]. In the case of supplements, the composition may also vary, and only in studies by a few authors, supplements were standardized, i.e., they contained a specific amount of the active substance [46,47]. So far, there have been studies involving chokeberry juice, but to the best of our knowledge, the effects of chokeberry fiber have not been assessed. Dietary fiber could help maintain metabolic health. Dose–response meta-analyses suggested an inverse association between the total fiber intake and the risks of cardiovascular, all-cause, and cancer mortalities [48]. Basu et al. (2021) have noted that supplementation with 280 g of whole blueberries and 12 g of soluble fiber daily for 36 weeks may prevent excess gestational weight gain, improve glycemic control, and reduce inflammation factors (CRP protein) in women with obesity. However, no effect on the lipid profile was observed [49]. In another study from 2023, it was observed that dietary fiber supplementation (24 g of dietary fiber powder/day) during pregnancy may prevent gestational diabetes mellitus and preterm birth in women before 20 weeks of gestation [50].

Our intervention assessed various anthropometric parameters. However, the influences of chokeberry fruit on the selected parameters are not clear. The mechanisms include decreasing lipogenesis and adipogenesis [51] and affecting cellular metabolism by modulating signaling pathways, including 5’-AMP-activated protein kinase [52]. Adipose tissue, especially visceral fat, plays an important role in the pathogenesis of metabolic disorders. Its excess, it contributes to the development of chronic low-grade systemic inflammation and, consequently, insulin resistance [53]. The body mass index (BMI) takes into account both the height and weight. A BMI score of 25–29.9 is overweight, while a score of 30 and above is obesity [54]. It is worth noting that BMI is not reliable because people with a high muscle mass can have a high BMI. In turn, skeletal muscles play an important role in maintaining metabolic health. This is mainly related to their participation in glucose uptake and increasing the sensitivity of cells to insulin [55]. In our study, no changes were observed in the bodyweight, BMI, fat tissue mass (kg), fat tissue percentage (%), or visceral fat (cm^2^) after 90 days of intervention. However, after 60 days of consuming only chokeberry juice, an increase in the muscle mass and a decrease in the waist circumference were observed. Then, after 30 days and adding chokeberry fiber—a decrease in the hip circumference was observed. In studies by other authors, no significant impacts of chokeberry juice consumption on anthropometric parameters (body mass, BMI, and waist circumference) were observed [56,57,58,59,60,61]. Only in a study by Kardum et al. (2014), there were observed decreases in the BMI and waist circumference in postmenopausal women with abdominal obesity after 4 weeks of chokeberry juice consumption [62]. Chokeberry juice and fiber do not seem to have significant impacts on anthropometric parameters. The overall diet and physical activity likely played a greater role, but chokeberry products may possibly support metabolic changes.

The next assessed parameters were the blood pressure and heart rate. Hypertension could be diagnosed when a person’s systolic blood pressure (SBP), in the office or clinic, is ≥140 mm Hg and/or their diastolic blood pressure (DBP) is ≥90 mm Hg, following repeated examinations [63]. Among the risk factors for hypertension, we can mention cardiovascular diseases, dyslipidemia, diabetes, a highly processed diet, smoking, alcohol consumption, and genetic predispositions, like a family history of hypertension [64]. The influence of chokeberry fruit on lowering blood pressure has already been documented. Hawkins et al. (2021), in a meta-analysis, have confirmed that chokeberry supplementation (both in the forms of juice and extract) can reduce systolic blood pressure [19]. Potential mechanisms include decreasing the oxidative stress and damage of the endothelium and reducing the activities of some factors, like endoothelin-1 and angiotensin-converting enzyme-1 [65,66]. Our study has also confirmed the beneficial effects of chokeberry juice consumption on the blood pressure—both systolic and diastolic. However, adding chokeberry fiber for 4 weeks did not enhance the antihypertensive effect. Other studies have shown positive effects for consuming juice in the amounts of 100–300 mL per day. The beneficial effects of the chokeberry juice on the blood pressure could be observed after just 4 weeks [59,62,67,68]. The exception was a study by Milutinović et al. (2019), in which no effects were observed [69]. In the case of the heart rate—initially, after 8 weeks, there was a decrease in this value and then the heart rate returned to the value at the beginning of the study. Comparing these results with those of other authors, Kardum et al. (2015) did not observe any impacts of chokeberry juice consumption on the 24 h pulse blood pressure or asleep pulse blood pressure, but they noticed a decrease in the awake pulse blood pressure [68]. Another study evaluated the heart rate using 30 mL of a standardized chokeberry extract, with the authors not reporting any observed changes [46].

Chokeberry may have a positive effect on carbohydrate metabolism, and anthocyanins probably could play the main role [70]. Several possible mechanisms are taken into account—including the protection of pancreatic ß-cells against oxidative stress [71,72], the effect on the increase in adiponectin, and the decrease in visfatin levels, which play roles in insulin sensitivity [73], increasing the degradation of glucose—alpha-amylase and alpha-glucosidase [74], impacting the activities of hepatic glucose metabolism enzymes [75], and inhibiting the activity of dipeptidyl peptidase IV (DPP IV) [74]. In a study from 2019 on mice with diabetes, a reduction in glucose levels was observed when the mice were given chokeberry juice. Chokeberry juice inhibited the activity of alpha-glucosidase in the upper part of the small intestine and the activity of DPP IV [76]. In another study from 2020, chokeberry extract given to rats with type 2 diabetes resulted in lower blood glucose and insulin levels. The mechanism responsible for this was the effects on liver enzymes involved in glucose metabolism—pyruvate kinase, phosphoenolpyruvate carboxykinase, glucose-6-phosphatase, and glucokinase [75]. The impact of chokeberry consumption by people on carbohydrate metabolism has been previously studied by other authors [59,62,67,68,69,77]. Changes in fasting glucose levels were noticed with the consumption of 150 mL of juice in studies by Gancheva et al. (2021) and Milutinović et al. (2019) for 12 weeks and with the consumption of 250 mL for 12 weeks in a study by Skoczyńska et al. (2007) [67,69]. Moreover, Gancheva et al. (2021) and Milutinović et al. (2019) observed decreasing HbA1c levels by drinking chokeberry juice [69,77]. In our study, there were reductions in the levels of glucose and glycated hemoglobin using smaller amounts of chokeberry juice, and the duration of the intervention was shorter. Importantly, extending this time to 12 weeks and adding fiber at the same time did not result in further reductions in glucose levels. In an earlier review [18], the authors hypothesized that a longer intervention time may be important, but this study does not confirm that conclusion. So far, insulin levels have not been tested in the case of chokeberry interventions involving people. High insulin levels may indicate developing insulin resistance in cells and an increase in hyperinsulinemia [78]. In studies assessing the effects of anthocyanins, it was shown that supplementation with purified anthocyanin (80 mg/capsule) could have beneficial effects on the homeostatic model assessment of insulin resistance (HOMA-IR) and adiponectin levels [79]. Our study did not show any important changes in insulin levels.

The positive impact of chokeberry consumption on the lipid metabolism may be based on several mechanisms, including reducing oxidative stress and pro-inflammatory factors, like TNF-alpha, or inhibiting the expression of specific genes, which are responsible for de novo lipogenesis, like fatty acid synthase [80,81,82]. In a study on intestinal CaCo2 cells, it was shown that the administration of chokeberry extract reduced the expression of genes involved in lipid metabolism, including sterol regulatory element-binding protein 1c, fatty acid synthase, and acyl-CoA oxidase 1 [82]. In other study, supplementation with anthocyanins derived from chokeberry improved blood lipid levels, had a beneficial effect on the intestinal microbiome, and regulated the AMP-activated protein kinase signaling pathway [83]. It has been shown that the activation of the AMPK pathway inhibits the enzyme 3-hydroxy-3-methylglutaryl coenzyme-A reductase, which reduces cholesterol biosynthesis [84]. Elevated levels of total cholesterol (TC), low-density lipoprotein cholesterol (LDL-C), and triglycerides (TG) are directly associated with increased cardiovascular risk and mortality [85]. In 2021, a meta-analysis was conducted and showed that daily supplementation with chokeberry for 6–8 weeks lowers total cholesterol levels. However, in this study, both chokeberry juice and chokeberry extract were taken into account. This impact was the most visible in people over 50 years of age [19]. In our study, the consumption of chokeberry juice also contributed to a decrease in TC, and the addition of fiber enhanced this effect. In studies by other authors, the effects of chokeberry juice on TC levels varied. Skoczyńska et al. (2007) noticed a decrease in the TC level after 12 weeks of juice consumption (250 mL) in people with mild hypocholesterolemia. Milutinović et al. (2019) also observed reductions in TC levels after 12 weeks in people with DM2 and on oral antidiabetic drug therapy for at least 6 months (150 mL of juice). The remaining authors did not notice any effect of the juice on the TC level. The duration of the intervention was 4–12 weeks, and the amount of juice was 100–300 mL [56,59,62,68,77]. The LDL-C level did not change significantly after 8 weeks of juice intervention, but there was a statistically significant decrease after the addition of the fiber. Similar conclusions were drawn by other researchers for consuming 100–200 mL of chokeberry juice over a period of 4–12 weeks [56,61,62,68,77]—LDL-C levels did not change. In turn, there are studies in which reductions in LDL-C levels have been observed—after consuming 150–200 mL of juice/day for 12 weeks [66,69]. It seems that despite the health-promoting properties of chokeberry juice, the overall diet is important in the case of LDL-C levels. Taking into account the impacts of this intervention on HDL-C levels, after 8 and 12 weeks of the intervention, the levels were lower than before its initiation. Other authors have demonstrated varied effects of chokeberry juice on HDL-C levels [56,59,62,69,77]. Kardum et al. (2014) and Milutinović et al. (2019) showed decreases in HDL-C levels after consuming 100–150 mL of chokeberry juice for 4–12 weeks [62,69]. Loo et al. (2016) and Gancheva et al. (2021), in contrast, did not observe any changes in HDL-C levels [59,77]—participants consumed 150–300 mL of juice for 8–12 weeks. Interestingly, in a study by Pokimica et al. (2019), the consumption of 100 mL of standardized chokeberry juice with a low polyphenol content reduced HDL-C levels, while juice with a high polyphenol content had no effect [56]. In the case of the triglyceride level, in our study, a downward trend could be observed after 8 weeks of consuming only chokeberry juice. Adding chokeberry fiber resulted in a return to preintervention values. Most studies conducted so far have not observed changes in TG levels [56,59,62,77]. In two studies involving juice, decreases in TG levels were noticed [67,68]. The homocysteine level did not change after 12 weeks of our intervention. In a study by Skoczyńska et al. (2007), a decrease in homocysteine was observed after a 12-week study using 250 mL of chokeberry juice [67]. Homocysteine is an amino acid that could be a risk factor for the development of cardiovascular diseases; therefore, its reduction may play an important role [86].

The liver has numerous necessary functions in the body, among which are the metabolism of proteins, lipids, and carbohydrates; detoxification processes; and the production of some substances, like bile [87]. Increasing liver enzymes, like alanine aminotransferase (ALT), aspartate aminotransferase (AST), and gamma-glutamyltransferase (GGTP), could be among the symptoms of liver diseases [88]. It is known that dietary antioxidants, including polyphenols, can improve liver function and reduce inflammation [89]. Polyphenols contained in chokeberry may improve liver functions by influencing the expressions of genes responsible for de novo lipogenesis in the liver (sterol regulatory element-binding protein, acetyl-CoA carboxylase, and fatty acid synthase); inhibiting proinflammatory factors, like TNF-alpha; and reducing oxidative stress [22,90,91]. In our study, the levels of AST and GGTP did not change after 8 weeks of juice consumption, but a decrease in the level of ALT was observed. Adding fiber after 8 weeks led to decreases in AST levels. The effects of juice consumption on liver functions have been assessed so far in three studies. Kardum et al. (2015) and Gancheva et al. (2021) did not observe any impacts of juice consumption on the levels of AST and ALT [68,77]. For the GGTP enzyme, Gancheva et al. (2021) reported a decrease, in contrast to Loo et al. (2016)—who observed no changes [59,77].

Uric acid is a product of the enzymatic breakdown of purine nucleosides and free nitrogen bases [92]. Elevated levels of uric acid are observed, among other things, in obesity, hypertriglyceridemia, hypertension, kidney diseases, and diabetes. There is also a relationship between hyperuricemia and the components of the metabolic syndrome [93]. However, uric acid could have antioxidant and pro-oxidative potentials [94]. The level of creatinine, in turn, can be a sign of the condition of the kidneys. However, creatinine values may fluctuate depending on the diet and protein intake, muscle mass, or some medications. Therefore, it is not the best diagnostic indicator because of its variability [95]. The glomerular filtration rate determines the amount of glomerular filtration in the kidneys. The GFR is usually estimated using equations based on concentrations of endogenous serum filtration markers, mainly creatinine. An e-GFR of ≥90 mL/min/1.73 m^2^ is considered as normal [96]. In our study, after 8 weeks of chokeberry juice consumption, there were increases in creatinine and uric acid and a decrease in the e-GFR index. The addition of fiber enhanced the decrease in creatinine. The impacts of chokeberry products on kidney functions have not been well researched, and so far, other authors have not confirmed the important role of chokeberry [67,68,69].

Inflammation is a part of the body’s defense mechanism. It is the process by which the immune system recognizes and removes harmful and foreign stimuli and begins the healing process. Inflammation can be either acute or chronic. Long-term inflammation is a pathological condition and increases the risk for developing many diseases and disorders, such as cardiovascular diseases, diabetes, cancer, and liver diseases [97]. We can assess inflammation in the body using various markers, including acute-phase proteins, TNF-alpha, and specific cytokines [98]. Because of its high antioxidant activity, chokeberry fruit may reduce inflammation [59]. The antioxidant and anti-inflammatory effects have been confirmed by studies on animals and cell lines [23,24,99,100]. In our study, there were no effects of the consumptions of chokeberry juice and fiber on CRP protein. Other authors also did not observe any changes in CRP levels [67,68,69]. The CRP level decreased in a study on overweight women after 12 weeks of consuming 150 mL of juice [77]. Not all studies confirm the beneficial effects of chokeberry on inflammatory markers. However, the effects of chokeberry seem to be promising because of its high antioxidant content, which is very important in the prevention and the treatment of many disorders.

The antioxidant potential of the plasma is our body’s ability to fight reactive oxygen species. One method for measuring this potential is the FRAP method [101]. The value of this potential is influenced by both endogenous antioxidants, such as antioxidant enzymes and uric acid, and exogenous antioxidants—supplied with the diet, including vitamins—vitamins C, E, and β-carotene; elements—magnesium, selenium, copper, manganese, zinc, and iron; and polyphenols, such as anthocyanins [7,102]. Chokeberry fruits have a high antioxidant potential compared to those of other fruits, which may translate to an increase in the body’s defense capabilities [103]. A meta-analysis from 2019 found that following a diet high in total antioxidants was associated with reduced risks of all-cause death, cardiovascular disease, and cancer [104]. In our study, there was a statistically significant increase in the antioxidant potential of the plasma after 8 and 12 weeks of intervention. Other studies did not assess the antioxidant potential of the FRAP, but the authors noted increases in antioxidant enzymes after the intervention with juice—there were increases in glutathione peroxidase [62] and superoxide dismutase [77]. However, the effect on the superoxide dismutase activity is not clear—in the study by Kardum et al. (2014) [62], this effect was not confirmed. The activity of the catalase did not change [62,77]. The consumption of chokeberry products may have a beneficial effect on the antioxidant capacity and, thus, increase the body’s defense mechanisms in the fight against excessive oxidative stress.

## 5. Conclusions

Our intervention has confirmed the positive impacts of the combination of chokeberry juice and chokeberry fiber consumptions on reductions in the systolic and diastolic blood pressures, heart rate, aspartate transaminase level, LDL cholesterol level, glycated hemoglobin level, glucose level, and waist circumference. After 90 days, increases in the antioxidant potentials of the serum were also observed. The impacts of this dietary intervention on other parameters seem to be ambiguous. This study’s findings suggest that these products may support metabolic health, but this hypothesis requires further research in the future.

## Figures and Tables

**Table 1:** Characteristics of the study group (*n* = 102).

Parameter	Av. ± SD	Min–Max	Med.	Q1–Q3
		Men (*n* = 35)		
Age (years)	45.9 ± 7.7	30.0–59.0	48.0	41.0–52.0
Weight (kg)	86.0 ± 15.9	61.8–120.0	88.6	71.9–97.5
Height (cm)	179.5 ± 7.2	161.0–188.0	181.0	173.0–185.0
BMI (kg/m^2^)	26.4 ± 4.1	19.6–34.4	26.5	23.3–29.2
		Women (*n* = 67)		
Age (years)	46.1 ± 8.9	31–64	46.0	38.0–53.0
Weight (kg)	71.5 ± 13.3	52.4–131.9	68.5	63.5–78.1
Height (cm)	165.4 ± 5.3	153.0–179.0	165.0	162.0–169.0
BMI (kg/m^2^)	26.1 ± 4.3	19.5–43.6	24.9	23.1–28.2
		Total (*n* = 102)		
Age (years)	46.1 ± 8.5	30.0–64.0	47.0	38.0–52.0
Weight (kg)	76.3 ± 15.7	52.4–131.9	71.7	64.9–84.5
Height (cm)	170.0 ± 8.9	153.0–188.0	168.0	164.0–176.0
BMI (kg/m^2^)	26.2 ± 4.2	19.5–43.6	25.3	23.2–28.4

Av.—average; Max—maximum; Med.—median; Min—minimum; Q1—lower quartile; Q3—upper quartile; SD—standard deviation.

**Table 2:** Dietary diary assessment during chokeberry intervention.

Stage of Intervention Study	Energy Intake (kcal)	Protein Intake (g)	Fat Intake (g)	Carbohydrate Intake (g)
Before Intervention	2084.88	90.32 (17.33%)	76.29 (32.93%)	265.71 (49.74%)
During Stage 2 of Intervention ^a^	2051.45	91.15 (17.77%)	78.31 (34.36%)	268.14 (47.87%)
During Stage 3 of Intervention ^b^	2063.93	89.46 (17.34%)	80.12 (34.94%)	270.48 (47.72%)

^a^—chokeberry juice consumption; ^b^—chokeberry juice and chokeberry fiber consumption.

**Table 3:** Compositions of chokeberry juice and fiber used for the dietary intervention.

Studied Component (Unit)	Content in Chokeberry Products	
	Chokeberry Juice	Chokeberry Fiber
FRAP (mmol/kg)	97.41	6.51
TPC (mg GAE/kg)	4566	765
Total Flavonoids (mg QE/kg)	791.70	63.70
Total Anthocyanins (mg Cy-3-GL/kg)	257.50	26.11
Vitamin C (mg/kg)	112.89	56.03
Magnesium (mg/kg)	222.02	990.88
Copper (mg/kg)	65.64	3.69
Iron (mg/kg)	1.07	60.31
Manganese (mg/kg)	6.49	38.61
Selenium (µg/kg)	26.86	212.91
Zinc (mg/kg)	1.15	17.26

FRAP—ferric-ion-reducing antioxidant potential; TPC—total phenolic content.

**Table 4:** Results of an intervention study involving chokeberry juice and fiber—data without statistically significant differences (*p* > 0.05).

Parameter	Before the Intervention, Stage “0”	After Stage 1 of the Intervention (Juice, 90 Days), Stage “1”	After Stage 2 of the Intervention (Juice + Fiber, Next 30 Days), Stage “2”
Weight (kg)	76.3 ± 15.7 (52.4–131.9) 71.7 (64.9–84.5)	76.4 ± 15.4 (52.2–133.4) 71.7 (64.9–84.9)	76.1 ± 15.5 (51.1–133.0) 71.6 (65.0–85.8)
BMI (kg/m^2^)	26.2 ± 4.2 (19.5–43.6) 25.3 (23.2–28.4)	26.1 ± 4.1 (19.4–44.1) 25.4 (23.2–28.0)	26.0 ± 4.0 (19.3–43.9) 25.3 (23.1–28.4)
Fat Tissue Mass (kg)	23.24 ± 8.90 (9.20–65.80) 21.65 (17.50–27.60)	23.12 ± 8.79 (6.60–67.00) 21.75 (17.90–27.40)	23.23 ± 8.84 (7.50–66.90) 22.30 (17.70–27.90)
Fat Tissue Percentage (%)	30.32 ± 8.01 (12.80–50.10) 30.90 (24.70–35.60)	30.19 ± 8.35 (10.20–50.20) 31.30 (24.70–35.60)	30.23 ± 8.24 (10.40–50.30) 31.25 (25.10–35.90)
Visceral Fat (cm^3^)	94.35 ± 45.50 (36.80–267.50) 81.75 (62.00–115.10)	96.01 ± 50.32 (30.20–304.20) 81.15 (62.80–122.30)	93.00 ± 45.56 (36.20–306.20) 80.90 (62.90–111.90)
Total Cholesterol (mg/dL)	213 ± 42 (88–337) 214 (186–240)	209 ± 39 (134–326) 212 (177–233)	207 ± 35 (122–303) 206 (180–230)
Triglycerides (mg/dL)	90 ± 38 (30–221) 82 (64–106)	88 ± 37 (38–205) 77 (61–105)	84 ± 34 (32–187) 78 (57–95)
GGTP (U/L)	24 ± 24 (5–162) 15 (13–26)	24 ± 23 (9–160) 17 (13–24)	24 ± 27 (11–188) 17 (13–25)
CRP (mg/L)	2.2 ± 2.4 (1.0–15.3) 1.1 (1.0–2.4)	2.1 ± 2.3 (1.0–12.0) 1.0 (1.0–1.7)	2.0 ± 2.8 (1.0–22.0) 1.0 (1.0–1.9)

BMI—body mass index; CRP—C-reactive protein; GGTP—gamma-glutamyltranspeptidase.

## Data Availability

All the data are available on request from the corresponding author.

## Supplementary Materials

The following supporting information can be downloaded at https://www.mdpi.com/article/10.3390/antiox13060699/s1: Table S1: Correlations between the FRAP and values of selected parameters, divided into research stages.
