# Supplementary material for: Consumption of Chokeberry Bio-Products Improves Specific Metabolic Parameters and Increases the Plasma Antioxidant Status
Source: Antioxidants (Basel). 2024 Jun 7;13(6):699. doi: 10.3390/antiox13060699 (PMC11200734; doi:10.3390/antiox13060699)
Supplement: Supplementary file 1 [file antioxidants-13-00699-s001.zip › antioxidants-3011638-supplementary.pdf]

antioxidants

MDPI

Supplementary Material

Consumption of Chokeberry Bio-Products Improves Specific Metabolic Parameters and Increasesthe Plasma Antioxidant Status

Ewa Olechno [1], Anna Puścion-Jakubik [2,*], Katarzyna Socha [2], Caterina Pipino [3], Małgorzata Elżbieta Zujko[1,*]

[1] Department of Food Biotechnology, Faculty of Health Science, Medical University of Białystok, Szpitalna 37 Street, 15-295 Białystok, Poland; ewa.olechno@sd.umb.edu.pl (E.O.); malgorzata.zujko@umb.edu.pl (M.E.Z.)
[2] Department of Bromatology, Faculty of Pharmacy with the Division of Laboratory Medicine, Medical University of Białystok, Mickiewicza 2D Street, 15-222 Białystok, Poland; anna.puscion-jakubik@umb.edu.pl (A.P.J.); katarzyna.socha@umb.edu.pl (K.S.)
[3] Center for Advanced Studies and Technology, G. d'Annunzio University, Chieti, Italy (C.P.)
* Correspondence: anna.puscion-jakubik@umb.edu.pl, malgorzata.zujko@umb.edu.pl

Table S1. Correlations between the FRAP and values of selected parameters, divided into research stages.

Parameter	R	*p*	R	*p*
	FRAP Stage 2		FRAP Stage 3	
Muscle mass	0.49	0.001	0.51	0.001
Waist circumference	0.45	0.001	0.48	0.001
Hip circumference	0.31	0.01	0.31	0.01
Systolic blood pressure	0.31	0.01	0.46	0.001
Diastolic blood pressure	0.28	0.05	0.42	0.001
Heart rate	ns	ns	ns	ns
Glycated hemoglobin	ns	ns	ns	ns
Glucose	0.26	0.05	0.32	0.01
Insulin	ns	ns	ns	ns
Creatinine	0.46	0.001	0.34	0.01
eGFR	ns	ns	ns	ns
Uric acid	0.87	0.001	0.78	0.001
HDL	-0.26	0.05	-0.41	0.001
LDL	ns	ns	ns	ns
AST	0.40	0.001	ns	ns
ALT	0.51	0.001	0.39	0.001
Homocysteine	0.36	0.001	ns	ns

ALT - alanine transaminase, AST - aspartate aminotransferase, eGFR - estimated glomerular filtration rate, HDL – high density lipoprotein, LDL – low density lipoprotein, ns - not statistically significant.
